# A Comprehensive Picture of Extracellular Vesicles and Their Contents. Molecular Transfer to Cancer Cells

**DOI:** 10.3390/cancers12020298

**Published:** 2020-01-27

**Authors:** Ancuta Jurj, Oana Zanoaga, Cornelia Braicu, Vladimir Lazar, Ciprian Tomuleasa, Alexandru Irimie, Ioana Berindan-Neagoe

**Affiliations:** 1 Research Center for Functional Genomics, Biomedicine and Translational Medicine, Iuliu Hatieganu University of Medicine and Pharmacy, 23 Marinescu Street, 400337 Cluj-Napoca, Romania; anca.jurj@umfcluj.ro (A.J.); oana.zanoaga@umfcluj.ro (O.Z.); cornelia.braicu@umfcluj.ro (C.B.); ciprian.tomuleasa@umfcluj.ro (C.T.); 2 Worldwide Innovative Network for Personalized Cancer Therapy, 94800 Villejuif, France; Vladimir.lazar@winconsortium.org; 3 Department of Hematology, The Oncology Institute Prof. Dr. Ion Chiricuta, 34-36 Republicii Street, 400015 Cluj-Napoca, Romania; 4 11th Department of Surgical Oncology and Gynaecological Oncology, Iuliu Hatieganu University of Medicine and Pharmacy, 400015 Cluj-Napoca, Romania; 5 Department of Surgery, The Oncology Institute Prof. Dr. Ion Chiricuta, 34-36 Republicii Street, 400015 Cluj-Napoca, Romania; 6 MEDFUTURE—Research Center for Advanced Medicine, Iuliu Hatieganu University of Medicine and Pharmacy, 23 Marinescu Street, 400337 Cluj-Napoca, Romania; 7 Department of Functional Genomics and Experimental Pathology, The Oncology Institute Prof. Dr. Ion Chiricuta, 34-36 Republicii Street, 400015 Cluj-Napoca, Romania

**Keywords:** cancer, extracellular vesicles, biogenesis, function, clinical implications

## Abstract

Critical processes such as growth, invasion, and metastasis of cancer cells are sustained via bidirectional cell-to-cell communication in tissue complex environments. Such communication involves the secretion of soluble factors by stromal cells and/or cancer cells within the tumor microenvironment (TME). Both stromal and cancer cells have been shown to export bilayer nanoparticles: encapsulated regulatory molecules that contribute to cell-to-cell communication. These nanoparticles are known as extracellular vesicles (EVs) being classified into exosomes, microvesicles, and apoptotic bodies. EVs carry a vast repertoire of molecules such as oncoproteins and oncopeptides, DNA fragments from parental to target cells, RNA species (mRNAs, microRNAs, and long non-coding RNA), and lipids, initiating phenotypic changes in TME. According to their specific cargo, EVs have crucial roles in several early and late processes associated with tumor development and metastasis. Emerging evidence suggests that EVs are being investigated for their implication in early cancer detection, monitoring cancer progression and chemotherapeutic response, and more relevant, the development of novel targeted therapeutics. In this study, we provide a comprehensive understanding of the biophysical properties and physiological functions of EVs, their implications in TME, and highlight the applicability of EVs for the development of cancer diagnostics and therapeutics.

## 1. Introduction

The tumor microenvironment plays a tremendous role in cancer development, especially in progression and metastasis. Bidirectional communication established between cells and their microenvironment is crucial for physiological and pathological conditions Such crosstalk occurs through cell-to-cell communication or the secretion of soluble factors, including chemokines, cytokines, and growth factors [1,2,3]. In the last decades, there has been an increasing interest in the implication of extracellular vesicles (EVs) involved in cell-to-cell communication. Many cell types secrete EVs, including dendritic cells [4], reticulocytes [5], lymphocytes [6], and cancer cells [7], and can be found in most body fluids [8]. Cell activation (platelet activation) causes the release of EVs together with modifications in pH, injury, hypoxia, irradiation, exposure to complement proteins and cellular stress [9]. Among them, blood clotting, stem cell differentiation, placental physiology, tissue regeneration, immunity and immunomodulation, reproductive biology, semen regulatory function, and pregnancy need to be underlined [10,11,12]. In this regard, EVs can also participate in pathological processes involving the progression of neurodegenerative disease and cancer [13]. According to their function, EVs mediate critical processes that underline cancer evolution, known as “hallmarks of cancer” [14,15], including inflammatory responses, cell proliferation, cell migration, invasion, immune suppression, angiogenesis, epithelial-to-mesenchymal transition, and metastasis [16,17]. Because EVs are involved in various processes responsible for cancer development and progression, these nanovesicles could become candidates as biomarkers and therapeutic tools against malignancies among other pathologies [10].

In our manuscript, we focus on the biogenesis and functions of EVs, exosomes, and microvesicles. Moreover, we described their content and their role in different biological processes and highlighted the applicability of the EVs for the development of cancer diagnostics and therapeutics.

## 2. EVs Classes, Biogenesis, and Content

**Figure 1:**
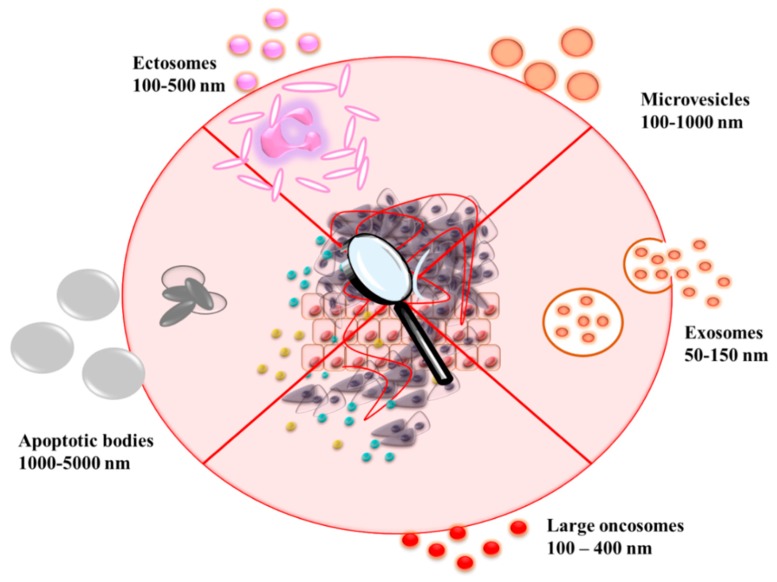
Various types of extracellular vesicles secreted from different cells, normal and tumor respectively.

EV is a global term used for all types of vesicles secreted by cells. EVs are classified according to their size, biogenesis process, and physical nature according to Table 1. The exosomes, the best characterized EVs, are generated by the internal budding of endosomes to produce multivesicular bodies (MVBs), which fuse with the plasma membrane releasing them in the extracellular space [18]. MVs are referred to as ectosomes or microparticles and formed by direct blebbing of the outward plasma membrane and released into the extracellular matrix. Another type of EV is the apoptotic body formed during cellular blebbing and fragmentation upon apoptosis [19]. Moreover, descriptions such as tolerosomes, prostasomes, epididymosomes, etc. [20], have been used to reflect the specific function of EVs or tissue-derived EVs (Figure 1).

### 2.1. Exosomes Biogenesis

In 1967, Peter Wolf investigated for the first time the aim of the platelet-derived vesicles highlighted their clotting properties [26]. A few years later, in 1981, exosomes were described for the first time by the Trams et al., which has shown that neoplastic cells release nanovesicles with 5′-nucleosidase activity [27]. In the next years, Johnstone et al. [28] showed that exosomes secreted in the extracellular space through the fusion between multivesicular bodies (MVBs) and the plasma membrane. Moreover, in 1996 was observed that B lymphocytes-derived exosomes exhibit antigen-presenting properties, allowing the induction of T-cell responses [29]. Also, Zitvogel et al. found that antigen-presenting exosomes derived from dendritic cells can suppress cancer progression [30]. Thereby, a comprehensive approach to exosomes’ functions provides a better understanding of various pathophysiological processes. Nanovesicles involved in regulating cancer development, neurodegenerative diseases, and a myriad of different other conditions are becoming increasingly important [31].

Exosomes are extracellular nanovesicles with a diameter ranging from 50 to 150 nm and are secreted by almost all cell types. Exosomes exhibit a characteristic cup-shaped morphology or are round vesicles, which are well delimited and analyzed through transmission and cryo-electron microscopy [32,33].

**Figure 2:**
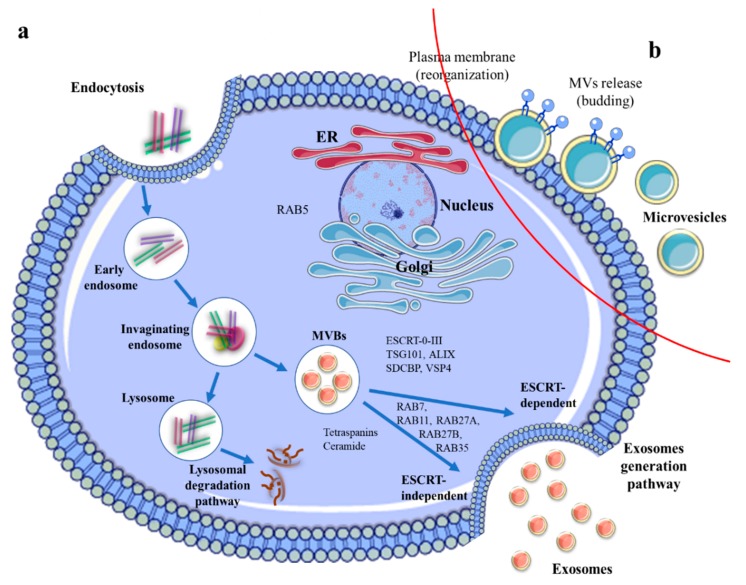
Biogenesis mechanism of (**a**) exosomes and (**b**) MV, and their release processes which are coordinated through two different intracellular pathways, such as exosomes generation pathway and lysosomal degradation pathway. The exosomes formation starts with an active process, called endocytosis, where the cells internalized the material in the extracellular fluid to form internal vesicles (early and late endosomes). Through the inward budding of the late endosomal membrane, multivesicular bodies (MVBs) are formed. Moreover, MVBs can fuse with the lysosomes where their content is degraded or can traffic and fuse with the plasma membrane to release the content into the extracellular matrix. The exosomes generation pathway can be regulated through ESCRT-dependent or via ESCRT-independent pathway. Both processes, (MVBs fusion with the plasma membrane and exosomes release) use for regulation Rab GTPases (Rab7A < Rab11, Rab27A, Rab27B, and Rab35) and SNARE protein complex.

The biogenesis and release of exosomes is a process controlled by endocytosis proteins and lipids. These processes start with the budding of an early endosome inner membrane, followed by the maturation of the multivesicular bodies (MVBs). After maturation, MVBs can be directed to lysosomes, entering the lysosomal degradation pathway, where their content is degraded by nucleases, proteases, lipases, and other hydrolytic enzymes from lysosomes lumen [34]. In this context, the MVBs, which fuse with lysosomes, display specific surface proteins, including tumor suppressor. His domain contains protein tyrosine phosphatase (HD-PTP), the HOP complex (HSP70-HSP90 proteins), the GTPase Ras-related protein Rab7A, and members of the membrane fusion proteins SNARE complex (soluble N-ethylmaleimide-sensitive factor attachment protein receptor) such as VAMP7 (vesicle-associated membrane protein 7), STX7 (syntaxin 7), and STX8 (syntaxin 8) [35]. The MVBs are integrated into the endosomal recycling system, where MVBs traffic and fuse with the cell membrane to release the exosomes in extracellular spaces. This process is under Rab guanosine triphosphate (GTPases) control, which includes Rab7A, Rab11, Rab27A, Rab27B, and Rab35 [36]. Alternatively, the exosome generation pathway can be regulated through ESCRT (endosomal sorting complex required for transport) complexes (referred to as the ESCRT-dependent pathway) or via an ESCRT-independent pathway. [37]. In the biogenesis process, ESCRT machinery is involved through the presence of four ESCRT multiprotein subcomplexes (ESCRT-0, ESCRT-I, ESCRT-II, and ESCRT-III), playing a tremendous role in facilitating MVB formation, vesicle budding, and protein cargo sorting [38]. Moreover, some supporting molecules, such as ATPase, ALG-2-interacting protein X (ALIX), or vacuolar protein sorting-associated protein (VPS4) are involved in the regulation of ESCRT-membrane scission machinery [37]. In addition, the ESCRT-independent pathway is orchestrated by neutral sphingomyelinases (a family of enzymes which are able to convert sphingomyelin in ceramide), as well as phospholipase D2 (PLD2) and ADP ribosylation factor 6 (ARF6) [10]. However, the formation and secretion of the exosomes are also controlled through the presence of several molecules, including [39] (p. 53) Rab27a, Rab27b, and syndecan-syntenin through its direct interaction with ALIX protein [40] (Figure 2a).

### 2.2. MVs Biogenesis

The biogenesis process for MVs is different and less known compared to exosomes biogenesis. MVs formation is an MVB-independent process and does not require exocytosis. In general, MVs have formed via direct budding of the plasma membrane through ARF6 and RHOA-dependent rearrangement of the actin cytoskeleton [41] (Figure 2b). ARF6-positive recycling vesicles are involved in trafficking cargo to the cell surface for incorporation in MVs [3]. Similar to exosomes biogenesis, ESCRT is also engaged in MV formation. Thus, TSG101 protein (tumor susceptibility gene 101) interacts with accessory proteins ALIX and ARRDC1 (arrestin domain-containing protein 1) during the last phase of MV release. ESCRT-III and ALIX were shown to be involved in cytokinetic abscission [42] and also Gag-mediated budding of HIV virions from cells [43].

Moreover, the activation of A-SMase (acid sphingomyelinase) stimulates the release of MVs from astrocytes and glial cells. MVs display unique lipid characteristics, including phospholipid phosphatidylserine (PS), which promotes uptake by recipient cells. Also, MVs are enriched in phospholipid lysophosphatidylcholine, sphingolipid sphingomyelins, acylcarnitine, and fatty acyl esters of L-carnitine. Ceramide and cholesterol have also been shown to play an essential role in MV formation [44].

MVs formation seems to be affected by several mechanisms. The extracellular concentration of calcium can affect MV structure because increased calcium levels induce membrane phospholipid scrambling and improve the formation of MVs. Moreover, in cancer pathology, calcium signaling is deregulated, and multiple tumor suppressors and oncogenes impact calcium regulation [45]. Hypoxia appears more often in solid tumors, which are associated with therapy resistance and tumor progression [46] and promoted MVs formation through a cellular process controlled by Rab22a and hypoxia-inducible factors (HIFs) [47]. Another mechanism responsible for MVs formation is actin deamination, where protein arginine deiminases (PADs) catalyze the hydrolysis of peptidyl-arginine to peptidyl-citrulline [48].

### 2.3. Apoptotic Bodies Biogenesis

Apoptotic bodies constitute a significant class of EV with various sizes (1000–5000 nm) formed exclusively during programmed cell death [49]. Apoptotic cells can go through critical morphological changes like membrane blebbing and membrane protrusion, releasing apoptotic bodies as a product of apoptotic cell disassembly [50]. Also, apoptotic bodies are functionally involved in the clearance of apoptotic cells by phagocytes [51] and modulate the response of the immune system [50]. The clearance of apoptotic bodies by macrophages executed via phagocytosis is mediated by interactions between phagocyte receptors and specific changes, like the oxidation of surface molecules in the composition of the apoptotic cell membrane [52]. Apoptotic volume decrease (AVD) is an early event occurring concomitantly with membrane blebbing and was also required for apoptotic body formation [53]. AVD starts within 0.5–2 h after apoptosis induction, where caspases are activated directly by death receptor ligation (extrinsic pathway) or mitochondrial dysfunction (intrinsic pathway) [54]. This process requires the efflux of osmolytes, mainly ions, via transporters and channels, followed by the simultaneous flux of water [55]. In this regard, it is thought that water extrusion of the cell is controlled by K^+^ Cl^−^ efflux and Na^+^ movements [53]. In this AVD process were described two distinct stages involved in cell death. The first stage of AVD takes place before cytochrome c release from the mitochondria, which determine cell volume decrease in 20–40% of cases. This stage is characterized by an electrolytic movement, leading to Na^+^ accumulation and K^+^ extrusion [56]. The second stage depends on the correct cytoskeleton organization and is also based on K^+^ extrusion. Therefore, both cytochrome c release and caspases are involved and essential for the AVD process [53].

Regarding the composition of these vesicles, they comprise a content including chromatin, small numbers of glycosylated proteins, large amounts of low molecular weight RNA and intact organelles such as mitochondria and nuclear fragments [27,57,58,59]. Recent studies have demonstrated that apoptotic bodies are involved in the progression and formation of the tumor microenvironment and metastasis [60,61,62]. Therefore, these vesicles can transfer bioactive molecules to “target” cells. For example, apoptotic bodies derived from mature endothelial cells stimulated the proliferation and differentiation of circulating endothelial progenitor cells [63]. Atkin-Smith et al. found that phosphatidylserine (PS) is the marker of apoptotic bodies [64] and phosphatidylserine (PS)-containing vesicles, including apoptotic bodies, increased tumor cell migration via the AXL receptor [65].

### 2.4. EVs Cargo Profiles

**Figure 3:**
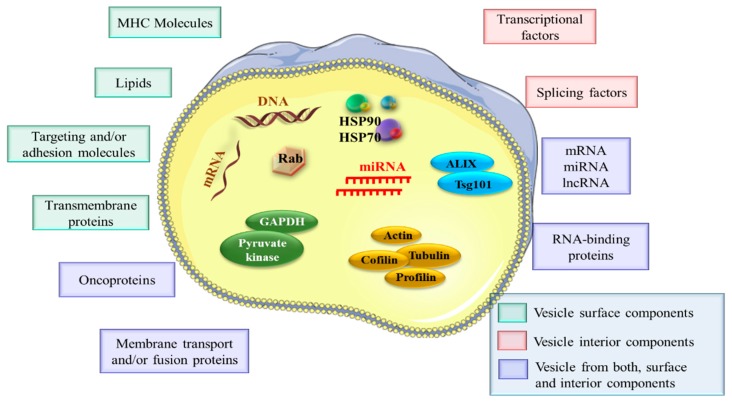
EV cargo profile. EVs deliver various bioactive molecules, including nucleic acids (DNA, mRNA, miRNAs (microRNAs), lncRNAs (long non-coding RNAs), specific proteins (oncoproteins), lipids, transcriptional factors, and RNA-binding proteins.

During the biogenesis process, EVs receive important bioactive molecules from their donor cells, including DNA (dsDNA, ssDNA, mtDNA), RNA (miRNA, rRNA, tRNA, snoRNA, snRNA, lncRNA, lincRNA or circRNA), lipids and specific proteins, including cytoskeletal proteins (actin, tubulin, cofilin, profilin), proteins associated with MVB formation (Alix, Tsg101) and enzymes (GAPDH, pyruvate kinase) (Figure 3). In general, these molecular constituents depend on cells of origin, environmental conditions, epigenetic changes, developmental stages and mechanisms of biogenesis. Nowadays, it is known that exosomes can contain different types of DNA such as single-, double-stranded genomic DNA and mitochondrial DNA [66]. The study performed by Balaj et al. showed that extracellular vesicles contain single-stranded DNA (ssDNA), which reflects the genetic status of tumor cells, including amplification of the oncogene c-Myc [67]. Kahlert et al. have shown that exosomes derived from pancreatic carcinoma cell lines contain long fragments of double-stranded DNA. Moreover, using genomic DNA from pancreatic cell line-derived exosomes, the mutations in KRAS and p53 were detected. Through whole-genome sequencing, it was observed that exosomes derived from serum patients with pancreatic cancer contain genomic DNA spanning all chromosomes [68]. These validated data by Thakur et al. revealed that tumor-derived exosomes contain double-stranded DNA predominantly located inside the exosomes [69].

**Figure 4:**
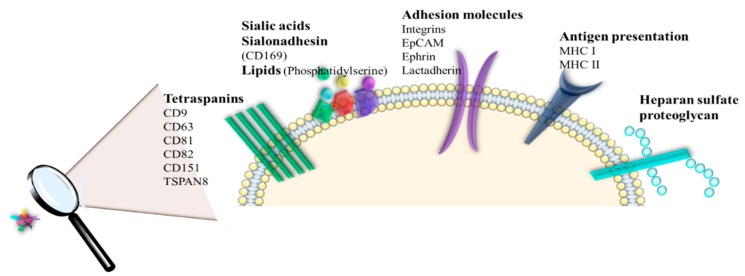
The presence of specific bioactive molecules on EV surface, which mediate the interactions between various ligands and receptors presented on the targeted cell surface (tetraspanins, adhesion molecules (integrins), lipids (phosphatidylserine), signaling receptors, molecules involved in antigen presentation and membrane trafficking (EpCAM—epithelial cell adhesion molecules, MHC—major histocompatibility complex).

Concerning the RNAs, many species of this class were found to be part of the exosome composition [70]. Specific proteins, including ESCRT complexes, ALIX, or Rab GTPases, which control exosomes biogenesis, are conserved and enriched in these nanostructures [71]. ALIX and TSG101 are also involved in exosomes release [72]. Heat shock proteins (HSP70 and HSP90 involved in antigen binding and presentation), tetraspanins (CD9, CD63, CD81, and CD82 take part in cell penetration, invasion, and fusion events) or integrins are packaged in exosomes content in order to participate in intracellular assembly and exosome trafficking (Figure 4) [31].

**Figure 5:**
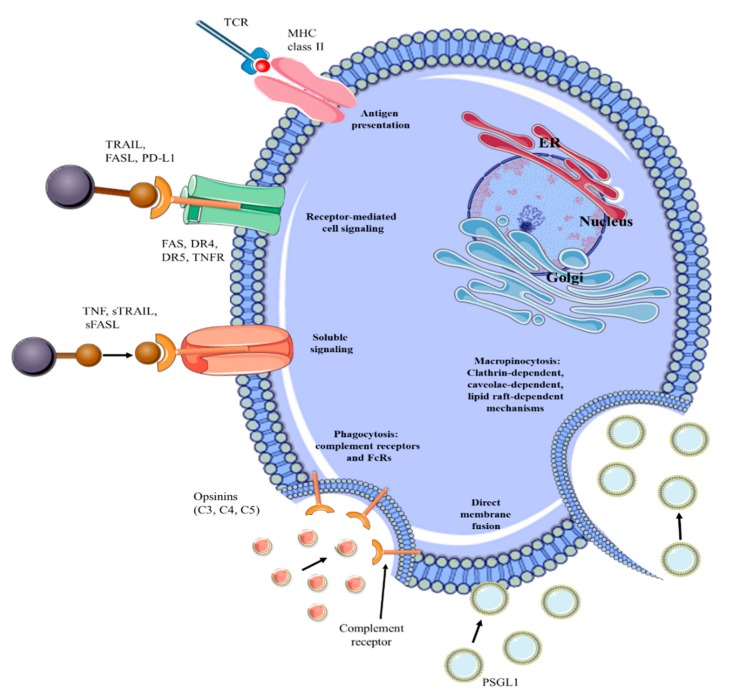
EV internalization by recipient cells through different mechanisms, including direct membrane fusion, macropinocytosis, endocytosis (clathrin- and caveolae-dependent mechanism, lipid-raft-dependent endocytosis) and phagocytosis (PI3K-dependent, dynamic-dependent, and actin-polymerization-dependent mechanism). The presence of ligand-receptors present on recipient cell surface can elicit biological responses and can targeted EVs (MHC—major histocompatibility complex, TCR—T cell receptor, TRAIL—TNF-related apoptosis-inducing ligand, FASL—FAS antigen ligand, PD-L1—programmed cell death 1 ligand 1, FAS—apoptosis-mediating surface antigen, DR4—death receptor 4, DR5—death receptor 5, TNFR—TNF receptor, sTRAIL—soluble TRAIL, sFASL—soluble FASL, C3—complement component C3, C4—complement component C4, C5—complement component C5, PSGL1—P-selection glycoprotein ligand 1, ER—endoplasmic reticulum).

Thus, exosomal protein content adds to the knowledge of pathophysiological activities of donor cells. The presence of the exosomal proteins including MET oncoprotein, mutant KRAS, and tissue factor are known to stimulate proliferation and coagulation, important biological processes involved in tumor formation [72]. The presence of integrins, ribonucleoproteins, RNA-binding proteins, and signaling receptors play an important role in the recognition of the targeted cell. Moreover, the abovementioned proteins are also involved in EV internalization in the targeted cell through several processes, including direct fusion, endocytosis (clathrin-dependent and caveolae-dependent mechanism, lipid raft-dependent, micropinocytosis and phagocytosis), lymphocyte-function-associated antigen 1 (LFA-1)-intercellular adhesion molecule 1 (AICAM1) and T cell receptor (TCR)-major histocompatibility complex (MHC) interactions [73] (Figure 5). The membrane composition of exosomes is highly abundant in specific lipid species, such as sphingomyelin, cholesterol, diacylglycerol, and ceramide [74]. Also, the formation and release of exosomes are regulated through the implication of some lipids and lipid-metabolizing enzymes (such as neutral sphingomyelinase (nSMase) or phospholipase D2 (PLD2)) [38]. Moreover, the extracellular microenvironment plays an important role in exosome secretion. In this regard, it was observed that the presence of an acidic extracellular pH causes te increased exosomes secretion as well as exosomal protein degradation. Meanwhile, alkaline pH reduces the secretion of exosomes and also exosomal protein and RNA. Thus, microenvironment pH plays a critical role in exosome secretion, and may influence their uptake into recipient cells [75].

### 2.5. Other (Additional) Extracellular Vesicles with a Complex Role in the Regulation of TME Components

#### 2.5.1. Ectosomes

The generation of ectosomes (diameter 50–500 nm) is different and not so well known compared to exosomes. The assembly of ectosomes required the accumulation of cargoes (partially different from those of exosomes) at the cytosolic surface of the plasma membrane. Thus, ectosomes are secreted through the budding of the outward cell membrane and released into the extracellular matrix [23]. This process may be due to the rearrangement of the asymmetric membrane consisting in a phospholipid layer induced by Ca^2+^-dependent enzyme, floppases, and flippases [76], and the action of ESCRT complexes which activate processes similar to those which take place during exosome generation [76,77]. During ectosome generation, additional factors regulate secretion into the extracellular matrix such as small GTPase ARF6 being active in vesicular traffic and the small GTPase of the Rho family (RhoA, Cdc42 and Rac1) which acts through the contraction of cortical actin under plasma membrane [23,78].

The composition of ectosomes is quite similar to that of exosomes. In ectosomes membranes, it can be observed a high level of cholesterol, sphingomyelin, and ceramide [79,80]. Any modification induced by Ca^2+^-dependent enzymes can determine the reorganization of phospholipid bilayers, where phosphatidylserine is translocated from the inner membrane to the outer membrane, followed by functional modification of the cytosol and plasma membrane. In this regard, phospholipid changes are attended by modifications in membrane proteins. Also, with the proteins present in exosomes structure, ectosomes consist of other proteins including the matrix metalloproteinase MT1-MMP, the adhesion protein P-selection, two glycoprotein receptors (GP1b and GPIIb/GPIIa) and the integrin Mac-1 [81]. The concentration of heat shock proteins and several enzymes in the ectosomes lumen is similar to those in the cytosol. Moreover, there are other luminal proteins, which are accumulated at high concentrations through various mechanisms. Thus, some proteins active in ectosome assembly are regulated by the ESCRT machinery and others by their direct interaction with the plasma membrane. The interactions between the specific proteins and the ectosomes lumen are mediated through the presence of protein anchoring, palmitoylation, myristoylation, sumoylation, high-order polymerization. Some proteins are found to be concentrated in deep areas of the ectosome lumen being directly bound by anchored proteins [82]. It was observed that Rho and Ras GTPases are involved in the control of ectosome cargo complexes assembly. Similar to the exosomes structure, the presence of miRNA, mRNAs and non-coding RNAs are abundant in ectosomes structure [58].

#### 2.5.2. Large Oncosomes

Among these nanovesicles, atypically large extracellular vesicles (0.5–5 µm in diameter) referred to as large oncosomes (LOs) have been identified. LOs can be byproducts of non-apoptotic cells when a large portion of the cellular membrane is shedding from the outward membrane during blebbing events [83]. The shedding process is induced by silencing of the cytoskeletal regulator DIAPH3 (Diaphanous-related formin-3) protein, by the overexpression of oncoproteins (Cav-1 (caveolin-1), HB-EGF (heparin-binding epidermal growth factor), MyrAkt1 (myristoylated Akt1)), or activation of the EGFR and AKT1 pathways [24,84,85,86,87]. These LOs carry abnormal and transforming macromolecules, including mRNA, microRNA, lipids, and biologically active proteins. LOs are enriched in a membrane-localized cytokeratin-18 and the expression levels of tetraspanins CD9, CD63, and CD81 are significantly reduced. As a protein enriched in large oncosomes, cytokeratin 18 has emerged as a potential marker that can be used to distinguish tumor-derived LOs from other types of EVs in plasma and tissues [24]. Also, the presence of Cav-1 in the LOs structure suggests the used of this as a marker for these extracellular vesicles. In some studies, the positivity of the Cav-1 in LOs structures can be used to detect metastatic disease in patients diagnosed with prostate cancer [24,88]. Interestingly, ALIX (programmed cell death 6 interacting protein) was observed also in LOs, even though this is a specific exosomes marker [87].

This unique composition allows LOs to operate as signaling devices, to send information to specific target cells and to modulate the primary and secondary tumor microenvironments [89]. It has been suggested that LOs have a vast repertoire of activities and may act as master regulators of various interactions networks which can promote processes such as tumor growth, inflammation, extracellular matrix remodeling, angiogenesis, inhibition of innate and adaptative immune response [89]. LOs can modulate tumor progression because they can degrade directly the extracellular matrix In Vitro. In this regard, LOs could release endothelial permeabilization factors that promote intravasation [90]. Moreover, through transferring RNA to endothelial cells, LOs may induce epigenetic changes and reprogram endothelial cells. In normal endothelial cells, LOs can induce migration, but tumor-derived LOs can facilitate endothelial leakage, allowing extravasation and colonization of distant sites [90]. Also, it was shown that LOs can export specific oncogenic cargo to other tumor or stroma cells. In this regard, the phenotype is reprogrammed by the oncogenic cargo establishing a tumor growth-supporting microenvironment [91].

#### 2.5.3. EV-Like Particles

This type of nanovesicles can be produced from virus-infected cells, such as retrovirus and Herpes virus. These EV-like particles are produced from the host cell plasma membrane and contain in their structure viral-gene encoded molecules [49] that do not generally lack a viral genome, making them non-infective [92]. Moreover, EVs is known as “gesicles” are derived from the Golgi organelle membrane and released from vesicular stomatitis virus (VSV) DNA transfected cells. In the structure of these EVs, VSV glycoprotein is found, which confers fusogenicity [93]. Also, non-infected cells can produce EVs derived from Golgi vesicles. In this regard, EVs contain specific proteins from Golgi and endoplasmic reticulum [94].

## 3. EVs Functions from the Beginning to the End of Their Route

The release of exosomes occurs at different periods of time after the biogenesis of ILVs. In many types of cells, MVBs which undergo exocytosis can operate as stores that prolong the release and delay the depletion of exosomes [23].

During the exosome’s biogenesis, MVBs move from the perinuclear cytoplasm transfer to the plasma membrane and fuse with it. Moreover, the control of the intracellular translocation and the fusion of MVBs is regulated through the involvement of some specific proteins including G proteins —Rab11, Rab27a and Ran2b [95,96]. The sites of fusion are dependent on the cells involved because this can be found across the plasma membrane or concentrated into a specific site. Thus, MVB fusion is regulated by a SNARE complex, which is composed of a P-SNARE with the MVB membrane and two Q-SNAREs (SNAP23 and syntaxin 1a) in the plasma membrane where the exosomes are released into the extracellular fluid [97].

Also, the release mechanism for ectosomes is different and much faster compared with the exosomes. Thus, once they are generated after the accumulation of specific molecules at their plasma membrane, ectosomes are immediately released into the extracellular fluid. Thus, in neurons and other neural cells, the release is governed by Ca^2+^ responses dependent on depolarization. On the other hand, in many other cells, ATP controls the release upon activation of its receptor P_2_X7. Moreover, the involvement of protein kinase C and ATP kinase in this process was highlighted. Also, in the presence of RhoA, Rac, and Cdc42 [98], the ectosomes release is preferred by contraction of cortical actomyosin close to the membrane microdomains [99]. The budding of the outward plasma membrane is followed by the production of spiral filaments by ESCRT-III and Vps4 ATPase, they induce membrane fission through a ring established at the budding neck. In the end, the scission of the plasma membrane and the final pinching off determine the release in the extracellular matrix of ectosomes vesicles [100].

It was found that a certain number of extracellular vesicles remain intact for a short period in the system until the membrane breaks down. Bioactive molecules (TGF-β, FGF, VEGF and other), which are contained in the structure of the vesicles, are released after the membrane breaks down. In this way, these molecules become available for the binding to their receptor on the targeted cells, following for the induction of specific responses [101]. Otherwise, some extracellular vesicles are resistant to membrane breakdown, having a long lifespan through the extracellular fluid. In the extracellular fluid, vesicles tend to accumulate close to intercellular junctions, moving through and between cells, to the adjacent areas of tissues [102] or blood serum, lymph, and cerebrospinal fluid. In this regard, the vesicles can bind to their specific target cells. It was observed that platelet-derived ectosomes bind monocytes and endothelial cells, and not neutrophils [103]; ectosomes bind mesenchymal cells, not mesenchymal stem cells, granulocytes, and endothelial cells [104]; ectosomes-derived neutrophils interact with platelets and also with macrophages and dendritic cells [105]. Moreover, exosomes derived from cortical neurons can bind only other neurons, not glial cells; exosomes-derived oligodendrocytes bind only microglia.

After the vesicles reach their target, they start rolling over the surface until they find sites for internalization. In the case of fibroblast-derived exosomes, when they reach their target, first they are recruited on filopodia and then start rolling until reaching a site on internalization [106]. For the internalization mechanism, molecules which are found on the outward plasma membrane play an important role. Thus, molecules such as tetraspanins, integrins, proteoglycans, and lectins seem to be involved in this process and are found in variable densities on vesicles, exosomes, and ectosomes respectively. In target cells, it was observed that the binding process is achieved through the presence of surface receptors, adhesion molecules, and two extracellular matrix proteins (laminin and fibronectin) [107]. After binding to surface receptors, vesicles induce signaling and they discharge their cargoes into the cytosol of target cells [108].

The initial interactions between EVs and target cells require high-affinity binding of at least two surface proteins, one from EVs and one from the plasma membrane of the target cell. It was observed that the protein of class I (syncytin-1 and syncytin-2) and class II (epithelial fusion failure 1 (EEF-1) protein) are possibly responsible for the binding and fusion of EVs. Thus, proteins of class I such as syncytin-1 and syntecyn-2 participate in the cell-to-cell fusion process [109]. These proteins are composed of α-helix-rich pre-fusion trimers which are responsible for insertion in the plasma membrane of their hydrophobic fusion peptides. After that, syncytin-1 and synticyn-2 refold as post-fusion trimers. Soluble carrier family 1 (ASCT-2) and major facilitator superfamily domain 2a (MFSD2a) are two transmembrane proteins implicated in the first step of exosome–membrane binding. These interact with syncytins on the surface of exosomes and are specific for exosomes caring carbohydrates for MFSD2a and neurotransmitters for ASCT2 [105]. Otherwise, protein class II includes epithelial fusion failure 1 (EEF-1) protein [110] which includes β-sheet-rich pre-fusion homodimers and heterodimers. These dimers include loops, which are inserted in the target plasma membrane. Moreover, the dimers refold into post-fusion trimers [111]. In the end, this process is unfolding through the insertion of the hydrophilic part of syncytins into the cell membrane, following by lipid reorganization, protein restructuring and membrane dimpling [105].

This process takes place in different locations on the plasma membrane surface. After this process occurs, the discharge cargo molecules are concentrated into the cytosolic layer near the surface of the cell [23] and start trafficking. Moreover, the initial binding process occurs at various endocytic sites. Once they get inside the cell, EVs are co-localize with lysosome markers, LAMP-1 for example, and following the endocytic pathway until their fusion with an endocytic membrane [112]. In this regard, the fusion of EVs with plasma or endocytic membranes induces the integration or discharge of its bioactive molecules into the cytosol, including nucleic acids, specific proteins, and lipids. These bioactive molecules may induce effects that may be stimulatory or inhibitory for target cells, induce changes of the gene expression, can significantly contribute to the turnover of many proteins, leading to the reprogramming of the target cell structure and function. EV cargoes are specific to parental cells and can modify the pathophysiology of the recipient cells, to activate various types of biological processes and to sustain cancer development [23].

## 4. EVs Role and Function

In the TME, cellular communication is a critical player of heterotypic and homotypic interactions, being involved in the tumor microenvironment changing [113]. Homotypic cell-to-cell adhesion is consisting of two or more cells from the same origin (for example, two tumor cells) or cells from different origin consisting of heterotypic cell-to-cell adhesion (for example immune cells inside tumor cells) [114]. Also, these small entities play an active role in tumorigenesis and metastasis, as well as response to therapy through the transfer of onco-miRNAs and oncogenes between tumor cells, and between tumor cells and TME [115].

### 4.1. Transfer of Homotypic EVs between Cancer Cells

One of the critical hallmarks of cancer is represented by the proliferation of tumor cells, process, which relies on soluble growth factors. Normal/tumor cells convey information to the TME through molecules packed in exosomes and other EVs via complex signaling networks [116].

The lipid bilayer membrane of the exosomes provides a protective shield that encapsulates and protects bioactive molecules, such as miRNAs or specific proteins from RNase degradation [117] or by proteinase [118]. Several studies have shown that the presence of the bioactive molecules contained in exosomes can modulate cell signaling events and biological processes of the targeted cells [119]. It was observed that, in established tumors, exosomes derived from glioblastoma can transfer functional mRNAs and miRNAs which stimulate tumor growth [120,121]. Moreover, between gastric cancer cells, lncRNAs are exchanged through exosomes which determine the progression of cancer [122]. In vitro, exosomes derived from gastric cancer cells can sustain proliferation in an AKT/PI3K and MAP kinase signaling-dependent manner [123]. With respect to exosomes secreted by breast cancer cell lines and patients with this pathology, it was demonstrated that exosomes contain miRNA-processing machinery which can induce transformation and tumor formation in non-tumorigenic breast cells [124]. Exosomes derived from tumor cells also can transport TGF-β (transforming growth factor-beta) from tumor cells to normal fibroblasts, TGF-β which initiate a program of differentiation of fibroblasts to a myofibroblastic phenotype [125]. Also, the exosomes can deliver bioactive molecules to distant cells. Some studies have investigated the exosomes derived from breast or pancreatic cancer-contained bioactive molecules, including telomerase [126] or macrophage migration inhibitory factor [127], and observed their role in the formation of premetastatic niches. Moreover, exosomes released by pancreatic cancer cells sustain cancer survival modifying the Notch-1 signaling pathway [128]. Through cell-to-cell communication, exosomes are involved in remodeling tumor microenvironments and the formation of the premetastatic niches in cancer development.

Other studies showed that exosomes derived from tumor cells can induce tumor cell proliferation in chronic myeloid leukemia [129] and gastric cancer [122], through PI3K/AKT and MAPK/ERK signaling pathways [123] respectively or lncRNA transfer [122]. Li at al. have shown that exosomal CD97 could mediate the signaling for tumor cell proliferation through the MAPK pathway [130]. Another study performed by Skog et al. observed that exosomes derived from glioblastoma induce cell proliferation in human glioma U87 cell line in a mechanism dependent on the CLIC1 protein [131].

Tumor-derived exosomes can modify the migratory status of targeted tumor cells. Exosomes derived from nasopharyngeal carcinoma were shown to improve the migratory capacity of the targeted tumor cells. This process is stimulated through the presence of epithelial to mesenchymal transition (EMT)-inducing signals (TGF-β, HIF1α, Annexin A2, Notch1 and LMP1 Casein Kinase II) in the exosomal cargos [116].

Therefore, exosomes can promote cancer metastasis and stimulate other cells. In this respect, tumor-derived exosomes induce epithelial–mesenchymal transition and matrix degradation, directly or indirectly disturb endothelial cells through macrophages activation, circulating tumor cells and the tumor activate platelets to release exosomes, influencing immune cells; exosomes can propagate cancer cells in a favorable niche to proliferate, which form a micro-metastasis, as well as the up-regulation of adhesive molecules on endothelial cells being stimulating by the tumor-derived exosomes [132].

Also, exosomes play a crucial role in tumor–tumor communication by transferring chemoresistance to a targeted tumor cell. This process was analyzed by Corcoran at al. when they reported that exosomes loaded with Docetaxel can transfer a resistance phenotype to prostate cancer cells, as well as in lung, liver and breast cancer [133]. Also, in lung cancer, it was observed that the transfer of Cisplatin resistance is regulated by exosomes containing a low level of miR-100-5p which increased the expression level of mTOR protein and chemoresistance in the target cells [134]. In breast cancer, it was observed that exosomes derived from drug-resistance cells contain different miRNAs that can modify the expression profile of specific target genes, PTEN (targeted by miR-222), Sprouty2 (targeted by miR-23a), p27 (targeted by miR-24) and APC4 (targeted by miR-452) [135]. Therefore, exosomal content can modulate the chemoresistance process in target cells that incorporate these exosomes and they can be used as non-invasive biomarkers in the oncology field for prognosis, diagnosis, and therapy.

### 4.2. Transfer of Heterotypic EVs in TME

**Figure 6:**
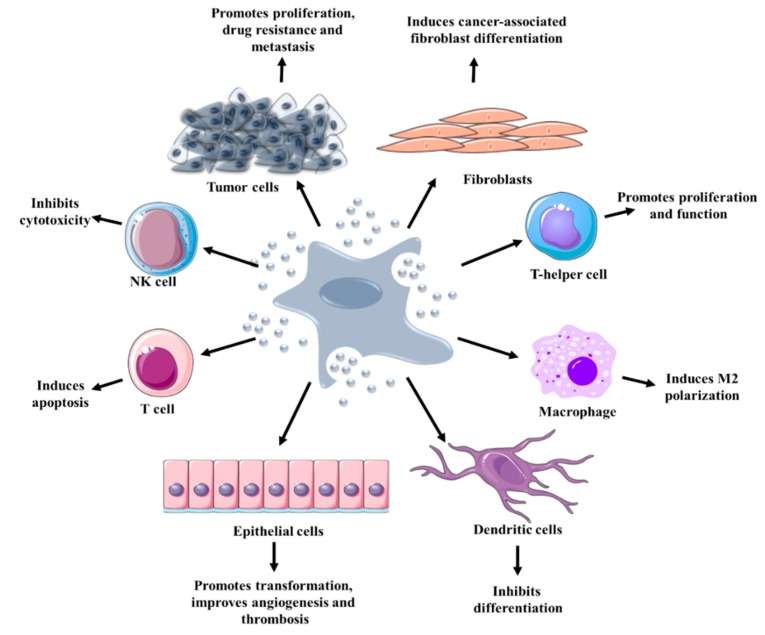
An overview of EV functions in TME cooperation. EVs derived from tumor cells act in an autocrine and paracrine manner. The interaction between tumor cells and other cells of TME through EVs may result in proliferation, tumor growth, metastasis, and drug resistance. EVs derived from tumor cells are involved in macrophage polarization, immune suppression, the transformation of fibroblast to cancer-associated fibroblasts, metastasis, induce cell death, enhanced angiogenesis, drug resistance.

The tumor microenvironment (TME) plays an important role and supports the growth, progression, and dissemination of a tumor. TME is a system organized of diverse cell types, including endothelial, fibroblastic, and immune cells, which are involved in and sustain all stages of cancer initiation, growth, and progression, and can adapt when challenged with therapies [136]. Moreover, the TME modulates the established interactions between cells through many signaling networks such as juxtacrine and paracrine interactions (exosomes, essential entities involved in paracrine interaction and involved in the mechanism of cell-to-cell communication) (Figure 6).

#### 4.2.1. Endothelial Cells

Endothelial cells play a tremendous role in tumor growth, invasion, angiogenesis and not only. The communication between endothelial cells and tumor cells promotes cell growth and is able to sustain drug resistance [137]. Through the expression and secretion of growth factors, including VEGF (vascular endothelial growth factor), TNF (tumor necrosis factor) and MCP-1 (monocyte chemoattractant protein-1) and hypoxia induction, tumor cells in association with some immune cells can promote the initiation of angiogenesis through the formation of leaky vessel structures and promote metastatic dissemination. Moreover, it was observed that miRNAs contained in exosomes derived from leukemia cells, miR-17-92 cluster, are involved in endothelial cell migration and maturation, which is common for cancer angiogenesis [138].

Exosomes that carry in their content epidermal growth factor receptor (EGFR) are involved in signaling pathways regulation of endothelial cells. In this regard, EGFR can activate the hepatocyte growth factor (HGF) through suppressing the expression levels of its upstream miR-26a/b [139]. Therefore, the up-regulation of HGF promotes gastric cancer as well as liver metastasis, while down-regulation of HGF suppresses liver metastasis [140]. TGF-β type II receptor is a common component in exosomes and can stimulate TGFβ signaling in the TME [141]. Exosomes derived from tumor-associated macrophages can target the miR-146b-5p/TRAF6/NF-kB/MMP2 pathway to suppress the migration process in endothelial cells [142].

#### 4.2.2. Fibroblasts

Fibroblasts actively support tumor cells due to their properties, including stress resistance, plasticity, cell-to-cell interactions, wound healing and fibrosis [136]. By influencing the microenvironmental secretome, which supports inflammation [143], controls immune recruitment [144], supports cancer-associated fibroblast (CAF) activation [145] and tumor proliferation, and enhances invasion and metastasis [146], the fibroblasts can promote tumorigenesis by TME modulation. Cancer invasion is supported by the presence of CAFs through the production of matrix metalloproteinase, which intensifies hypoxic conditions [147] and reshapes the extracellular matrix of the TME [148].

Due to their content, exosomes are an essential component of fibroblast and cancer cell signaling. It was observed that leukemia cells-derived exosomes accelerate CAF activation to remodeling the TME and also extracellular matrix to adopt a more cancer-permissive state [149]. Exosomes derived from prostate CAFs stimulate TME metabolism to a glycolytic, less oxidative profile which is usually common for solid tumors by improving glutamine metabolism, reducing the level of mitochondrial function and being a source of intermediate metabolites. Moreover, CAF-derived exosomes regulate the metabolism of tumor cells through inhibiting the mitochondrial oxidative phosphorylation process [150]. In colorectal cancer stem cells, it was demonstrated that exosomes isolated from CAFs cells that developed a drug resistance mechanism can induce and accelerate drug resistance through paracrine signaling [151]. Therefore, exosomes secreted from fibroblasts can increase the migratory capability of breast cancer cells through the activation of the WNT-signaling pathways [152]. Also, this type of exosome contains mtDNA, which activates oxidative phosphorylation that leads to endocrine therapy resistance [153].

#### 4.2.3. Immune Cells

In the TME, immune cells are crucial players, which assure defensive protection against any foreign antigens (microbes, viruses, toxins, and cancer cells). This collection of cells secrete cytokines, chemokines, growth factors, and proteolytic enzymes, which mediate tumor progression, actively kill tumor cells, and modulate immune evasion [154]. The selection and migration of immune cells into TME are controlled by dynamic signaling [155] and exosomes [156]. Exosomes can directly activate the immune cells, including natural killer (NK), B and T cells, and macrophages [21]. It was observed that exosomes can act as immune-suppressors where they can hinder the cytotoxic activity of effector CD4 and CD8 T cells and NK cells [21], and inhibit the differentiation of dendritic cells (DCs) [157,158]. According to this correlation, it’s fair to consider that RNA and miRNAs are responsible for the activation or suppression of the innate and adaptive immune systems. In a study, it was shown that exosomal RNA can activate myeloid cell population [159]. In this regard, the role of RNA in this process is understudied compared to miRNAs. However, some studies have investigated the role of miRNAs as toll-like receptor (TLR) ligands in different types of cancer which stimulate the progression of cancer cells [160,161].

The communication between immune system cells is mediated by the involvement of critical factors. For example, exosomal LFA-1 contributed to the communication between dendritic cells and T cells. Integrins, which are found on exosomes outer membrane derived from B-cells, are specifically involved in the selection of cell populations with which they will interact. Moreover, the presence of phosphatidylserine (PS) in the bilayer membrane of exosomes can mediate the uptake by the PC receptor on the dendritic cells and macrophages [162].

In some studies, it is shown that exosomes loaded with specific antigens and peptides are capable of sustaining the activation of CD4^+^T cells and CD8^+^T cells, even in the absence of dendritic cells. More interestingly, after presenting antigens, exosomes derived from dendritic cells stimulate CD4^+^T cells and CD8^+^T cells and participate in the activation of natural killer cells in an antigen-specific manner [163]. Exosomes secreted by virus-infected macrophages can stimulate the secretion of TNF-α by macrophages and neutrophils [164].

Natural killer (NK) cells can release exosomes in both resting and activated conditions [165]. Moreover, released exosomes express both typical NK markers (CD56) and killer proteins (FASL and perforin) and also exert antitumor and immune homeostatic activities. FASL and perforin from exosomes are released by resting NK cells. In this regard, exosomes derived from NK cells also have control over the immune cell expansion only upon activation through cell-extrinsic or cell-intrinsic stimuli [166].

Macrophages, an important key for the tumor microenvironment, are responsible for, and mediate various processes, including cancer-related inflammation, matrix remodeling, immune escape, and cancer metastases [167]. According to the microenvironment, macrophages can acquire various functional phenotypes. In this regard, macrophages are classified according to their polarized phenotypes into classically activated macrophages (M1) and alternatively activated macrophages (M2). M1 macrophages produce proinflammatory cytokines to eliminate infectious microbes and sustain the Th1 immune response [162]. M1 macrophages are usually formed In Vitro by interferon-γ and/or the stimulation with lipopolysaccharide (LPS). On the other hand, M2 macrophages present an anti-inflammatory phenotype, participate in parasitic infections and T-helper 2-type immune responses, and sustain tissue remodeling and wound healing. In vitro, M2 macrophages are regulated by interleukin-4 (IL-4) and/or stimulation of IL-13 [168].

The study performed by Garzetti et al. found that RNA transcripts, which are specifically sorted and packed into EVs, reflect the phenotype of M1 and M2 macrophages [169]. Moreover, Ismail et al. showed that EVs with a size range between 0.05–1 µm and released from activated macrophages can induce the differentiation of naive monocytes into activated macrophages. After validation, they also observed the expression of macrophage lineage markers (CCR5, CD16, CD206) and an increased level in the phagocytic activity. Also, they showed that the presence of miR-233 in the EVs seems to be a major player that induced the differentiation in the monocyte [170]. McDonald et al. observed that LPS-activated RAW264.7 macrophages released EVs that carried ten different proinflammatory cytokines [171]. However, for a comprehensive understanding of exosome function in immune systems, further investigations are needed.

#### 4.2.4. Cancer Stem Cells

Cancer stem cells (CSCs) are involved in cancer initiation and metastasis and were found to release exosomes that mediate cellular communication by miRNAs delivery [172]. Cancer stem cells derived exosomes (CSC-exo) can act as potent tumor microenvironment regulators [173]. For example, exosomes derived from glioma stem cells (GSCs) play an important role in the control of tumor immunity acting as mediators of cell-to-cell communication [174]. Also, GSCs-exo can deliver genetic information and proteins in the tumor environment by internalization in brain microvascular endothelial cells (MVECs) [131]. Moreover, exosomes from GSCs with up-regulated levels of miR-26a can be used as therapeutics in glioma [172]. miR-21 was shown to be overexpressed in GSC-derived exosomes and can be delivered to endothelial cells (ECs) stimulating the angiogenic ability of these cells [175]. Also, endothelial cells were activated by exosomes obtained from renal cancer stem cells enhancing tumor vascularization [176].

CD90+ liver CSC released exosomes can mediate the upregulation of VEGFR1 in endothelial cells and stimulate angiogenesis via H19 lncRNA [177]. Tumor stroma modulation mediated by CSC-exo is the most important characteristic of exosome signaling in cancer cells [178]. CSC-specific signaling proteins like beta-catenin was also exported in exosomes. Exosomes contain CSC-specific molecules including phenotypic surface receptors (CD133, CD44), stem cell factors (Oct4), functional enzymes (ALDH) and transcriptional activators-Notch pathway components (Jagged 1, Dll4) [179].

## 5. Clinical Utility of EVs

Nowadays, the Holy Grail in cancer area is represented by the discovery of biomarkers used for early detection. Specifically, a useful biomarker should be specific for a certain tumor type, to detect the pre-metastatic stage and support the use of non-invasive techniques. Regarding liquid biopsies, the focus is on circulating tumor cells, cell-free tumor RNA, cell-free tumor DNA and more interesting, recently, exosomes gained attention in precision or personalized medicine [180]. Due to their content, liquid biopsies provide clinical information before and during treatment and help to improve the therapeutic plan and monitoring. EVs secreted by tumor cells seem to be a reliable source of cancer-associated molecules with potential as biomarkers for different types of cancer, although there is a long way before they are clinically applicable because of the need for more information and the difficult process involved in exosome isolation [181]. EVs contain a vast repertoire of bioactive molecules, which makes them potential biomarker candidates. In addition to miRNAs, bioactive molecules such as oncogenic mRNAs (fuse gene and splice variant transcripts), lncRNAs, double-stranded DNA fragments (cancer driver mutation genes) and lipids provide a comprehensive understanding regarding the selected pathology and gained attention as potential biomarkers [10].

### 5.1. EVs in Drug Resistance

Drug resistance in cancer treatment has been observed to be a major obstacle. Recent studies have demonstrated the role of exosomes as mediators in drug resistance through a drug efflux-dependent mechanism which allows the elimination of the therapeutic agent from the cancer cells [37,182]. Also, exosomes may transfer specific miRNAs in sensitive recipient cells changing their chemo-susceptibility by modulating drug-induced apoptosis, signaling pathways, cell cycle distribution and gene expression [183,184].

Plasma membrane transporters like P-glycoprotein (P-gp) were observed to play an important role in drug efflux in the aqueous phase. For instance, doxorubicin was encapsulated and exported by tumor-derived exosomes [185]. Exosomes can mediate cisplatin efflux in human ovarian cancer cell lines participating in the process of tumor resistance [186]. In colon cancer cells, miR-34a and miR-145, CRC-derived exosomal miRNAs were shown to contribute to cell proliferation and the induction of drug resistance [187], while miR-192 and miR-215 induced chemoresistance to fluoropyrimidine and antifolates [188,189]. Wnt activity and drug resistance were increased in differentiated CRC cells by exosomal Wnts both In Vitro and In Vivo [190].

Studies on different types of cancer found that cross-talk of cancer cells is initiated by stromal cells via exosomes [191,192,193]. Thereby, a new strategy was discovered for paclitaxel resistance in ovarian cancer cells represented by miR-21 exosomal transfer prevention from stromal cells [191]. In the exosomes transfer process from stromal cells to breast cancer cells was observed activation of antiviral retinoic acid-inducible gene 1 enzyme (RIG-1) signaling was observed, which regulates the proliferation of drug-resistant cells [193]. The decreasing of antibodies may be explained by the exosome’s modulation of their binding in cancer cells. For example, in breast cancer cells, derived- exosomes expression of HER2 was found to be increased and to interfere with monoclonal antibody trastuzumab activity [194]. A powerful exosome release was observed in aggressive B-cell lymphoma modulated by ATP-binding cassette (ABC) transporter A3 (ABCA3), leading to exosome-mediated target cell protection as a major factor of tumor cell susceptibility to antibody therapy [195].

Multiple drug resistance was correlated with a significant increase of drug transporters expression from the adenosine triphosphate (ATP)-binding cassette transporter (ABC) family [44] which are proteins using energy from ATP hydrolysis for drugs removal from cells and for preventing anti-cancer drugs accumulation [196]. The expression of multidrug resistance protein 1 gene (MDR1, ABCB1) was found to encode p-glycoprotein in 50% of cancers with MDR phenotype and was induced by chemotherapy [197], causing therapy resistance of the recipient cells [198,199].

On the other hand, ectosomes seems to be involved in multidrug resistance (MDR) and remain an obstacle in cancer chemotherapy. Recent studies showed that MDR phenotypes are transferred to sensitive cells also via ectosomes. Moreover, ectosomes released by MDR chronic/acute myeloid leukemia cells modified the phenotype of sensitive cells through to transfer of functional P-gp or MRP1 and the mRNAs for both proteins [200]. Within the ectosomal cargo, some of the abovementioned transporter proteins are transferred alongside cytoskeletal proteins, CD44 and ERM (moesin, ezrin, radixin) protein family [201]. In a study by de Souza et al., it was observed that upon incubation of ectosomes with different inhibitors of apoptosis (IAPs), cells became more resistant to cell death when treated with cisplatin and paclitaxel [202].

More interestingly, ectosomes may also facilitate the expulsion of chemotherapeutics from tumor cells and sustain their survival. In this regard, the human breast cancer cell line MCF-7 treated with doxorubicin accumulated and released the chemotherapeutic drug in shed microvesicles [185]. Thus, ectosome-releasing agents are considered as potential alternative drugs in differentiation therapy against acute myeloid leukemia (AML). Anso-Adda et al. showed that stimulation of promonocytic leukemia cells (THP-1) with phorbol myristate acetate, histamine, and all-trans retinoic acid increased the amount of ectosomes released by these cells. Moreover, released ectosomes, which contain TGF-β1 can inhibit the proliferation of THP-1 cells and they induce differentiation of those cells to macrophages/ monocytes [203].

### 5.2. EVs as Biomarkers

Exosomes are biocompatible and biodegradable extracellular vesicles with low toxicity and the ability to deliver endogenous biological cargo to specific targets over a long distance [204] and soluble drugs across the blood–brain barrier [205] (Table 2). The bilipid membrane of exosomes forms a protective shield and increases the cellular internalization for the encapsulated anti-cancer drugs [206]. Exosomes originating from autologous cancer cells caused minimal toxicity during transport to target cells, reach parental cancer cells through endocytosis, increase the cytotoxicity of these cells [207], and can be less immunogenic than artificial delivery vehicles [206]. Different drugs, such as doxorubicin, paclitaxel, docetaxel, or polyphenols like curcumin, were encapsulated in exosomes [208,209,210]. Both in vitro and in vivo studies have shown antitumor activity in the case of the doxorubicin delivery platform using exosomes [211]. Kim et al. validated a paclitaxel delivery platform with exosomes using a sonication procedure. Thus, a vector moiety was incorporated with AA, a ligand with a high affinity for sigma receptor, and the obtained AA-PEG-exoPTX formulation showed a strong capacity to accumulate in target cancer cells [212].

Exosomes were also observed to deliver siRNA and miRNA direct against oncogenes [213,214]. In vivo studies found that vessel density was lower in mice tumors with siRNA delivered by exosomes compared with mice treated with free siRNA [215]. In pancreatic cancer cells, siRNA delivery by exosomes derived from fibroblast was higher than liposomes delivery [204]. miRNA cargo delivery by exosomes was present in different types of cancer cells: exosomes expressing the EGFR binding molecule GE11 in breast cancer xenografts [216], miR-122 in hepatocellular tumors [217], miRNA-143 in osteosarcoma cells [218], and miR-146b to glioma cells [219].

Generally, ectosomes contribute to the pathogenesis of different types of cancer and can be used as biomarkers for diagnosis and prognosis as well as surveillance indicators for cancer patients. In some types of cancer, the ectosomes levels are significantly increased in glioblastoma [220], multiple myeloma [221] and non-small lung cancer [222] compared with those of healthy patients. Moreover, was observed that ectosomes levels in colorectal carcinoma patients are significantly reduced. On the other hand, ectosomes can be used to distinguish benign tumors from malignant breast [223] and prostate [224] cancer but were not observed between benign colorectal disease and colorectal carcinoma [225].

In patients with malignant breast cancer, it was observed that ectosomes exhibited an increased expression of several surface antigens (CD66, Her2/neu, BRCP, Hsp27) compared with benign tumors [223]. On the other hand, in colorectal and pancreatic cancers ectosomes expressed surface glycoproteins such as MUC1 (mucine1), CEA (carcinoembryonic antigen) and CA19-9 (carbohydrate antigen 19-9). More interesting, the expression of the abovementioned surface glycoproteins is different between the pathologies. Thus, in colorectal cancer, the expression of MUC1 is higher and CA19-9 exhibits an elevated expression in pancreatic cancer [224]. Moreover, the presence of ectosomes in peripheral blood provides a novel prognostic tool to monitor malignant cells in multiple myeloma, due to an increased number of CD138 positive cells which have been correlated with tumor burden [221].

### 5.3. EVs as Cancer Therapeutic

In tumor cells, exosome secretion was observed to be accompanied by an alteration of local and systemic tumor environments and to induce tumor growth progression, metastasis, and lack of sensitivity to drugs. For this reason, a functional method for cancer therapy may be created by the destruction of exosomes dissemination pathways through tumor cells or by the removal of exosomes from the circulatory system [206]. Studies on the effect of tinzaparin, an antithrombotic drug, in human pancreatic carcinoma cells revealed that cell migration was significantly inhibited, with tinzaparin leading to tissue factor pathway inhibitor (TFPI) release from cancer cells which blocks PAR-activating TF complexes [235].

Exosomes derived from different sources may have a fairly wide range of applications in cancer therapy. For example, exosomes derived from dendritic cells (DCs) can be used as anticancer vaccines due to the nature of DCs as antigen-presenting cells (APCs) [236]. CD86 was found to be expressed on the surface of DC-derived exosomes [4,237] and their administration after incubation with cancer-antigen induced cancer-specific T cell response [238]. Further, in vivo studies by co-culturing CD40 exosomes with DCs found that exosomes trigger high antitumor immunity inducing DCs maturation and stimulating tumor antigen-specific CD4+ T cell proliferation [239]. Phase I and II clinical trials performed on NSCLC patients revealed the preference of exosomes to stimulate T cell- and NK cell-based immune responses in cancer patients [240]. Adipose-derived stromal cells (ASCs) derived exosomes can be used in the therapy of prostate cancer due to the fact that exosomal miR-145 reduced Bcl-xL activity and induced prostate cancer cell apoptosis via caspase-3/7 pathway [241]. In Vitro studies demonstrated that human amniotic epithelial cell-derived exosomes may restore ovarian function in chemotherapy-induced premature ovarian failure (POF) mouse model by transferring miRNAs [242]. Jessian et al. found that the anti-miR-9 delivered from bone marrow (BM)-MSCs to glioblastoma multiform (GBM) via exosomes reversed P-glycoprotein (P-gp) expression and sensitized GBM to Temozolomide (TMZ) [243]. Therefore, exosome-based treatment can be a great alternative in cancer therapy by the disruption of tumor cell homeostasis, immune response activation, and delivery of chemotherapeutic agents.

Ectosomes can carry a vast repertoire of bioactive molecules by offering a unique carrier system to deliver different types of therapeutic agents to cancer cells, enabling local drug delivery. In this regard, Tang et al. have shown that malignant hepatocarcinoma cell line, H22, incubated with methotrexate, doxorubicin or Cisplatin released ectosomes loaded with chemotherapeutic drugs [244]. It was observed that in vitro, isolated ectosomes exhibited a cytotoxic effect on tumor cells and in vivo reduced hepatocarcinoma and ovarian cancer growth. Thus, chemotherapeutic agents encapsulated into ectosomes showed higher efficacy and fewer adverse effects compared with free drug administration.

Ectosomes also have an increased potential as agents for virotherapy, being able to transfer an oncolytic adenovirus into the nucleus of tumorigenic cells. The experiments performed in vitro and in vivo showed the fact that this delivery system is fatal to tumor cells and reduced tumor growth in vivo in adenocarcinoma mice. Further, ectosomes loaded with this oncolytic adenovirus seem to be more efficient compared to the free virus [245].

miRNAs also represent other promising bioactive molecules that can be transferred via ectosomes and regulate target gene expression and functions of recipient cells (Table 3) [246].

## 6. Conclusions

In this paper, we have focused on characterization of EVs, their biogenesis and function, and most relevant, their clinical implications in the oncology field. Moreover, exosomes and microvesicles which are different in their origin, distribution, cellular lifetime, mechanism of release, and association, are remarkable molecules that exhibit tremendous information about cancer pathologies. These EVs are constantly released from many types of cells, normal or pathological, and develop significantly, from their navigation through the extracellular fluid to interact with target cells, binding/fusion/integration with plasma membranes, to the discharge of cargo after uptake, and are involved in cellular phenotype. Moreover, it is known that EVs are important mediators in various biological processes and can establish cell-to-cell communication. This cellular function is induced through the exchange of large molecules, including various types of RNA, proteins and, in some cases, DNA. Moreover, EV coordinated cell networks are a critical component in various cancers and are involved in disease spreading and metastasis.

An important role in cell biology is played by the accumulation of molecules during EV assembly. In the biogenesis process, EVs are enriched with specific proteins and lipids that are specific for donor cells. In this regard, upon release and fusion, EV components induce significant modifications in recipient cells, interfering with the expression levels of some specific proteins and other molecules. In many cancer cells, EVs are involved in the control of various and critical biological processes through the content of their specific bioactive molecules.

## Figures and Tables

**Table 1:** The classification of extracellular vesicles and their main characteristics.

Types of Extracellular Vesicles	Size [nm]	Appearance by Electron Microscopy	Markers	Genetical Information	Mechanism of Information	Release Process	Pathways	Lipid Membrane Composition	Protein Components	Intracellular Origin	References
Exosomes	50–150	Cup shape	CD63, TSG101, Alix, flottlin, tetraspanins, Rab5a/b, HSP70, HSP90	DNA, non-coding RNA, miRNA	Multivesicular bodies fusion with plasma membrane	Constitutive and/or cellular activation	ESCRT-dependent, tetraspanins-, ceramide-, stimuli- dependent	Enriched in cholesterol, sphingomyelin, ceramide, lipid rafts, phosphatidylserine	Tetraspanins (CD9, CD63, CD81, CD82), Multivesicular body biogenesis (ALIX, TSG101)	Endosomes	[21]
Microvesicles	100–1000	Irregular shape	Integrin, selectin, flittilin-2	mRNA, miRNA	Outward blebbing of the plasma membrane	Constitutive and/or cellular activation	Ca^2+^ - dependent, cell- and stimuli-dependent	Expose phosphatidylserine, enriched in cholesterol, diacylglycerol, lipid rafts	Cell adhesion (integrins, selectins), death receptors (CD40 ligands)	Plasma membranes	[22]
Ectosomes	100–500	Bilamellar round structures	β1 integrins, selectins, CD40, MMP, lineage markers, erzin	mRNA, miRNA	Outward blebbing of the plasma membrane	Constitutive and/or cellular activation	Ca^2+^ - dependent, cell- and stimuli- dependent	Enriched in cholesterol, diacylglycerol, phosphatidylserine	Enzyme (proteolytic enzymes)	Plasma membranes	[23]
Large oncosomes	100–400	Heterogeneous	Cytokeratin-18, CD9, CD63, CD81, Cav-1	mRNA, miRNA	Outward blebbing of the plasma membrane	Constitutive and/or cellular activation	EGFR & AKT pathways, silencing of the cytoskeletal regulator DIAPH3 by ERK	Phospholipid phosphatidylserine	Cytoskeleton components (cytokeratin 18), tetraspanins (CD9, CD81), cell adhesion molecules (integrin-α3, integrin-αV, ICAM, CD44)	Plasma membranes	[24]
Exosome-like vesicles	20–50	Irregular shape	VSV-G	mRNA, miRNA	From Golgi organelle membrane		Overexpression of VSV glycoprotein	Do not contain lipid rafts	Growth factors and cytokine (TNFR1)	Internal compartments	[25]
Apoptotic vesicles	1000–5000	Heterogeneous	Histones, DNA, Annexin V	mRNA, miRNA, DNA	Programmed cell death and cell shrinkage	Apoptosis	Apoptosis-related	Phosphatidylserine	Transcription and protein synthesis (histones)	Plasma membrane, cellular fragments	[22]

TSG101: tumor susceptibility gene 101; DNA: deoxyribonucleic acid; ESCRT: The endosomal sorting complexes required for transport; HSP: heat shock protein; RNA: ribonucleic acid; mRNA: messenger RNA; miRNA: microRNA; MMP: matrix metalloproteinase; Cav-1: caveolin-1; EGFR: epithelial growth factor receptor; ICAM: intercellular adhesion molecule 1; ERK: the extracellular-signal-regulated kinase; DIAPH3: diaphanous related formin 3; VSV-G: vesicular stomatitis virus G protein; TNFR1: tumor necrosis factor receptor 1.

**Table 2:** Potential clinical applications for exosomes in various types of cancer.

Exosomes Applications	Type of Cancer	Marker in Exosomes	Remarks	References
**Diagnosis**	Colorectal	Circulating exosomes in plasma	The highest number of plasma-derived exosomes were associated with tumor differentiation and overall survival	[226]
		miR-21, miR-23a, miR-150, miR-1229, miR-1246	In tumor patients, the level of those miRNAs was upregulated	[227]
	Gastric	linc00152	A higher level in plasma of patients with gastric cancer	[228]
	Pancreatic	GPC1 protein	Use as specific marker for early diagnosis	[229]
	Prostate	Circulating exosomes in plasma	The highest number of plasma-derived exosomes were associated with tumor differentiation and overall survival	[224]
**Drug delivery**	Cancer cells which developed MDR	PTX	exoPTX are suitable for the delivery of various chemotherapeutics to the drug resistance cancer cells.	[230]
**Treatment**	Bladder cells	Heparin	Inhibit exosomes uptake in bladder cancer cells	[231]
	Hematopoietic cell	Calcium	Regulator of exosomes biogenesis	[232]
	Pancreatic cells	Gw4869	Biogenesis inhibition of cancer cell derived exosomes - blocks exosomes oncogenic roles	[192]
**Immunotherapy**	Melanoma	Dendritic cells	Exosomes derived from dendritic cells have positive effects on patients with these pathologies	[233]
	NSCLC	Dendritic cells		[234]

miR: microRNA; GPC1: glypican 1; MDR: multidrug resistance; PTX: paclitaxel; NSCLC: non-small cell lung carcinoma.

**Table 3:** EVs as drug delivery agents for cancer therapy.

Therapeutic Agents	Cancer Type	Ev Source	Target Cell	Remarks	References
**Small molecules**					
**Paclitaxel**	Prostate	Prostate cancer cell lines, LNCaP and PC3	Prostate cancer cell lines, LNCaP and PC3	EVs isolated from the prostate cancer cells previously treated with Paclitaxel, increased the cytotoxicity of the drug in vitro against autologous prostate cancer cells.	[207]
	Pancreatic	Murine SR4987 MSCs	Human pancreatic cell line, CFPAC-1	EVs loaded with paclitaxel in vitro cancer cell proliferation.	[247]
**Doxorubicin**	Lung	Human lung cancer cell lines, H1299 and YRC9	Human lung cancer cell lines, H1299, A549, MRC9—lung fibroblast, HCASM—smooth muscle cells	Inhibited cancer cell growth in vitro.	[248]
	Breast and ovarian	Human breast cancer cell line, MDA-MB-231, and mouse ovarian cancer cell line, STOSE	MDA-MB-231 and STOSE (used for In Vitro experiments and also injected into mice)	In vitro: presented cytotoxicity against cancer cells. In vitro: reduced tumor volume and cardiotoxicity compared with free doxorubicin.	[249]
**Paclitaxel and doxorubicin**	Brain	Brain endothelial cells, bEND.3	Human brain neuronal glioblastoma—astrocytoma U-87MG xenograft in zebrafish	EVs delivered anticancer drug the blood-brain barrier to xenograft transplanted brain cancer cells. Reduced expression levels of VEGF RNAs compared with free drugs.	[250]
**Cisplatin**	Lung	Tumor cells previously treated with chemotherapeutic drugs	Hepatocarcinoma cells—resistant murine H22, human breast cancer cell line, MCF-7, human lung cancer cell line, A549	Extracellular vesicles released from tumor cells containing cisplatin reversed drug resistance and induced apoptosis in drug resistance tumor cells derived from patients with lung cancer.	[251]
**AO**	Melanoma	Macrophages	Melanoma cell line, Me30966	Exosomes isolated form macrophages which contain AO increased apoptosis in melanoma cell line compared with free AO in vitro.	[252]
**siRNAs**					
**VEGF siRNA**	Brain	Brain endothelial cell line, bEND.3	Human brain neuronal glioblastoma- astrocytoma U087MG xenograft in zebrafish	VEGF siRNA contained in exosomes crossed the blood-brain barrier to xenograft transplanted brain cancer cells to decrease tumor burden in vivo.	[253]
**miRNAs**					
**Let-7**	Breast	Human breast cancer cell lines, HCC70, HCC1954 and MCF-7, expressing GE11 (a peptide which targets EGFR)	In Vitro—human cell line, HCC70. In Vitro—EGFR-expressing breast cancer xenograft tissue in Rag^2-/-^ mice	Exosomes which contain GE11 targeted EGFR that is expressed in cancer tissues and inhibited tumor growth compared with cancer tissue that contain non-let-7 and exosomes containing non-GE11 in vitro.	[216]
**miR-122**	Liver	Human AMSCs	In Vitro—human liver cancer cell line, HepG2 In Vivo—injected mice with HepG2 cell line	In vitro, exosomes that contain miR-122 in their structure induced apoptosis in liver cancer cell and decreased tumor growth in mice compared with exosomes which do not contain miR-122.	[217]
**Anti-miRNAs**					
**Anti-miR-9**	Glioblastoma	Human mesenchymal stem cells	Human glioblastoma cell lines, U87 and T98G and glioma cell lines isolated from patient, BT145 and BT164	In vitro, exosomes that contain anti-miR-9 reversed the resistance of glioblastoma cells to temozolomide compared with exosomes which does not contain anti-miR-9.	[243]
**mRNAs**					
**CD-UPRT**	Schwannoma	Human embryonic kidney cell line, HEK293T	In Vivo—HEI-193 cells in a Schwannoma orthotopic mouse model	In Schwannoma, mRNA of CD-UPRT-containing exosomes induced apoptosis and in vivo have the ability to reduced tumor growth via increasing 5-fluorocytosine sensitivity compared with the exosomes which does not contain CD-UPRT.	[254]
**Proteins**					
**TRAIL**	Myeloma	Human chronic myelogenous leukaemia cell line, K562	In Vitro—human multiple myeloma cell lines, SUDHL, INT12 and KMS11 In Vivo—SUDHL and INT12 cells injected into mice	In vitro: exosomes containing TRAIL have potential to induce myeloma cell death In vivo: reduced tumor growth in mice more efficient than free TRAIL	[255]
**hMUC1**	Colon	Mouse colon cancer cell line, CT26 and TA3HA, which expressed hMUC1	MUC1-expresseing mouse cell lines in BALB/c nude mice In Vitro—BMDCs from BALB/c mice	Exosomes that contain hMUC1 stimulated splenocytes through promoting IFNγ release In Vitro and In Vivo having the ability to suppress tumor growth compared with non-hMUC1 exosomes.	[256]

EV: extracellular vesicle; MSCs: mesenchymal stem cells; VEGF: vascular endothelial growth factor; RNA: ribonucleic acid; mRNA: messenger RNA; siRNA: small interfering RNA; miRNA: microRNA; AO: Acridine Orange; EGFR: epidermal growth factor receptor; CD-UPRT: cytosine deaminase fused to uracil phosphoribosyl transferase; TRAIL: TNF-related apoptosis-inducing ligand; IFNγ: interferon gamma.
